# Land-use change is associated with multi-century loss of elephant ecosystems in Asia

**DOI:** 10.1038/s41598-023-30650-8

**Published:** 2023-04-27

**Authors:** Shermin de Silva, Tiffany Wu, Philip Nyhus, Ashley Weaver, Alison Thieme, Josiah Johnson, Jamie Wadey, Alexander Mossbrucker, Thinh Vu, Thy Neang, Becky Shu Chen, Melissa Songer, Peter Leimgruber

**Affiliations:** 1 Trunks and Leaves Inc., 82 Wendell Avenue, STE 100, Pittsfield, MA 01201 USA; 2 Conservation Ecology Center, Smithsonian’s National Zoo and Conservation Biology Institute, Front Royal, VA USA; 3 Department of Ecology, Behavior and Evolution, University of California, San Diego, La Jolla, CA USA; 4 Environmental Studies Program, Colby College, Waterville, ME USA; 5 School of Environmental and Geographical Science, University of Nottingham Malaysia, Kuala Lumpur, Malaysia; 6 Frankfurt Zoological Society, Jambi, Indonesia; 7 Department of Wildlife Management, Vietnam National University of Forestry, Hanoi, Vietnam; 8 Wild Earth Allies, Phnom Penh, Cambodia; 9 Zoological Society of London, London, UK; 10 United States Department of Agriculture Agricultural Research Service, Beltsville, MD USA; 11 College of Science, Health, Engineering and Education, Murdoch University, Perth, WA Australia

**Keywords:** Conservation biology, Ecological modelling, Biogeography, Environmental impact, Biodiversity

## Abstract

Understanding historic patterns of land use and land cover change across large temporal and spatial scales is critical for developing effective biodiversity conservation management and policy. We quantify the extent and fragmentation of suitable habitat across the continental range of Asian elephants (*Elephas maximus*) based on present-day occurrence data and land-use variables between 850 and 2015 A.D. We found that following centuries of relative stability, over 64% (3.36 million km^2^) of suitable elephant habitat across Asia was lost since the year 1700, coincident with colonial-era land-use practices in South Asia and subsequent agricultural intensification in Southeast Asia. Average patch size dropped 83% from approximately 99,000–16,000 km^2^ and the area occupied by the largest patch decreased 83% from ~ 4 million km^2^ (45% of area) to 54,000 km^2^ (~ 7.5% of area). Whereas 100% of the area within 100 km of the current elephant range could have been considered suitable habitat in the year 1700, over half was unsuitable by 2015, driving potential conflict with people. These losses reflect long-term decline of non-forested ecosystems, exceeding estimates of deforestation within this century. Societies must consider ecological histories in addition to proximate threats to develop more just and sustainable land-use and conservation strategies.

## Introduction

Habitat loss and degradation are leading drivers of terrestrial biodiversity loss worldwide^1–3^. An estimated three quarters of the Earth’s land surface has been significantly altered by human activities^4^. Historic reasons include conversion for cultivation and settlement, reflecting both local and global socioeconomic drivers of land-use and land-cover (LULC) change^5,6^. Climate change is an additional contributor to species declines within the past century^4,6^. As a result of these anthropogenic changes to climate and land-use, global forest extent is estimated to have been reduced by 32% relative to the pre-industrial period and ecological communities are estimated to have lost over 20% of their biodiversity^4,6^.

Although LULC trends in recent decades may be inferred from satellite imagery and statistical data^2,7,8^, it remains difficult to assess the impact of long-term anthropogenic processes on particular species or ecosystems. Human-induced changes are known to restrict the ranges of many terrestrial mammal species^9,10^ but historical records on population abundance and distribution are often limited for many taxa, complicating efforts to assess impacts over longer periods. Nevertheless, longer historical perspectives are necessary to appreciate the true magnitude of changes to threatened ecosystems. For example, historical studies have influenced conservation policies related to remnant prairies in Oregon, wetlands in Iowa, and forests in Germany, at times challenging standard management practices^11^.

**Figure 1:**
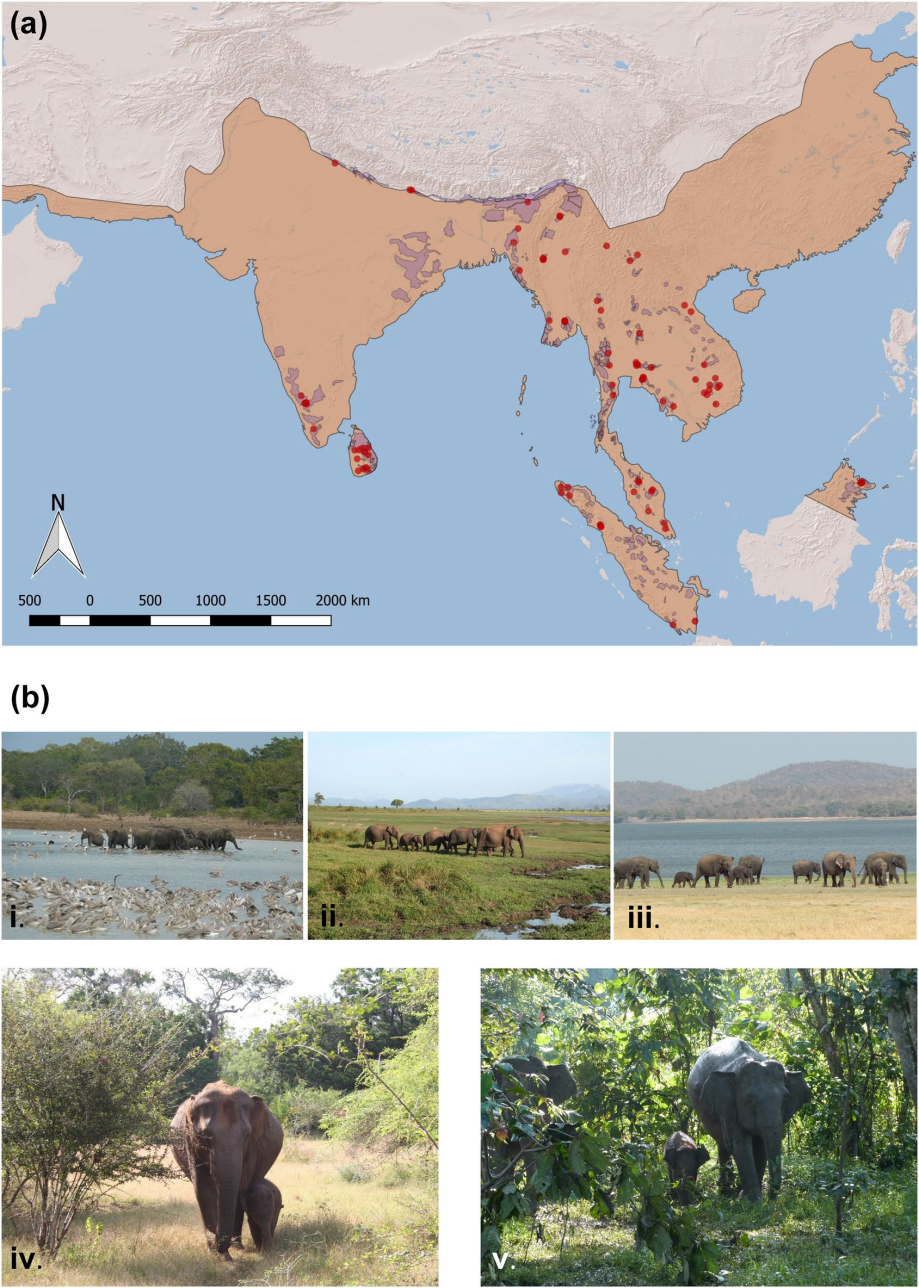
Locations of elephant occurrence. (**a**) Brown shaded region shows presumed historic post-glacial range (Olivier^20^), smaller purple polygons show current range (classified as “active confirmed” in Hedges et al.^85^), points show sampled occurrences. Map created by S. de Silva in Quantum GIS (QGIS, https://www.qgis.org) v.2.18.25. (**b**) Examples of Asian elephant ecosystems. Upper panel: anthropogenic water sources of varying ages and scale at which elephants gather in Sri Lanka. (**i**) A small reservoir originally built and maintained at village-level with inhabitants resettled in the 1980s after the creation of Udawalawe National Park, now maintained by wildlife managers. (**ii**) A large dammed reservoir completed in the 1970s and maintained by the national government, the impetus for creation of Udawalawe National Park. (**iii**) The large Minneriya reservoir built by King Mahasen in the third century and restored in the 1800s following British occupation. Small reservoirs provide year-round water whereas large reservoirs also yield floodplain vegetation for forage. Lower panel: Asian elephants occur in dry seasonally deciduous forests (**iv**) as well as lush a-seasonal rainforests. Photos: S. de Silva.

One way to overcome these data gaps is through ecological niche models (see also species distribution models), in which species occurrence data, together with environmental covariates, are used to infer possible occurrence or suitable habitat at a different area or time^12–14^. We model historic range in suitable habitat over the past 1165 years for a widely-distributed endangered mammal, the Asian elephant (*Elephas maximus*). Elephants are ecosystem engineers that uniquely influence the structure of ecosystems^15^. Asian elephants inhabit ecosystems ranging from grasslands to rainforests on Earth’s most densely populated yet biodiverse continent, representing diverse ecoregions^16^ rather than particular biomes (Fig. 1). Elephants also exemplify mutual challenges for people and wildlife at frontiers of land-use change that manifest as “human-elephant conflict” (HEC)^17,18^. Asia contains up to 70% of the world’s indigenous human population^19^, along with several hotspots of species decline and threatened megafauna^9^. Modelling changes in suitable habitat for elephants, therefore, indicates the transformation of a wide range of ecosystems consequential for both wildlife and human communities.

The extant Asian elephant range (Fig. 1) is thought to represent only a subset of the species’ historic range^20^, with the far western population thought to have gone extinct by 100 BCE and most of those in mainland China (with the exception of some southern provinces) extirpated by the fourteenth century BCE^21^. Published depictions of historic elephant range are based on limited historical and anecdotal accounts; however, elephants were probably never distributed uniformly across such a wide area^22^. Therefore, the extent of change in available habitat, necessary for species assessments such as that of the IUCN Red List^21^, have hitherto relied on rough estimates. Moreover, the dominant paradigm of elephant management across Asia has typically been to simply drive (or translocate) elephants into forests, especially those designated as sanctuaries, with little consideration of habitat availability or dispersal requirements^23,24^.

We characterized change in the extent and fragmentation of elephant ecosystems for the period between 850 and 2015 A.D using land-use variables from the Land-Use Harmonization 2 dataset^25^ (hereafter LUH2) and an ecological niche model constructed with the maximum entropy (MAXENT) algorithm. Our aims were to: (1) quantify historic trends in the extent and fragmentation of suitable habitat for elephants throughout Asia at a broad spatial scale, (2) characterize the suitability of present-day elephant range, (3) identify areas of potential habitat outside these ranges, and (4) characterize regional vulnerabilities for extant populations based on historic trends and considerations for long-term sustainability. We emphasize the need to appreciate long-term landscape histories in order to understand present-day distributions and the needs of both elephants and people in the future.

## Results

### Historic changes

**Figure 2:**
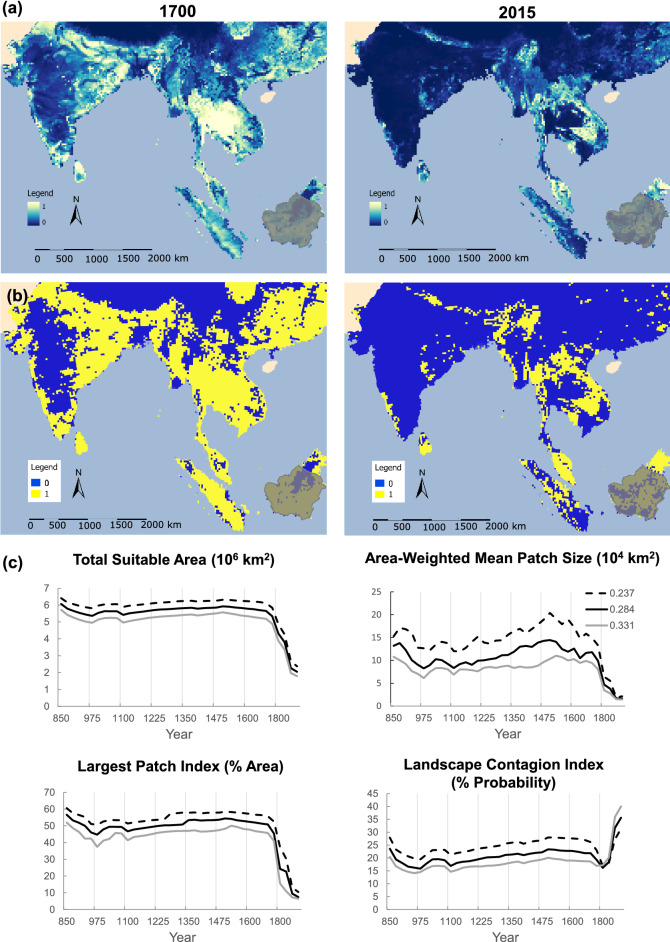
Loss of suitable habitat from 850 to 2015. Masked areas (Hainan Island and part of Pakistan) have been excluded from analyses, and for visual clarity all of China is not shown. Shaded area (Borneo) is outside the currently known historic range. (**a**) Habitat suitability predicted on the basis of elevation and the Land-use Harmonization (LUH) variables from the year 2000. (**b**) Binarized map where 1 (yellow) indicates “suitable” areas with values above 0.284 (threshold of ‘maximum training sensitivity plus specificity’) and 0 (blue) indicates “unsuitable” areas. (**c**) Changes in the extent and spatial configuration of suitable habitat, where each curve corresponds to the given threshold value. Total Suitable Area is the sum of all suitable habitat across the range. Area-Weighted Mean Patch Size is the weighted average of patch sizes. The Largest Patch Index is the percentage of total area occupied by the largest patch. The Landscape Contagion Index can be thought of as a measure of homogeneity, with higher probabilities representing fewer, more clumped patches. See Table S4 for complete list of fragmentation measures.

**Figure 3:**
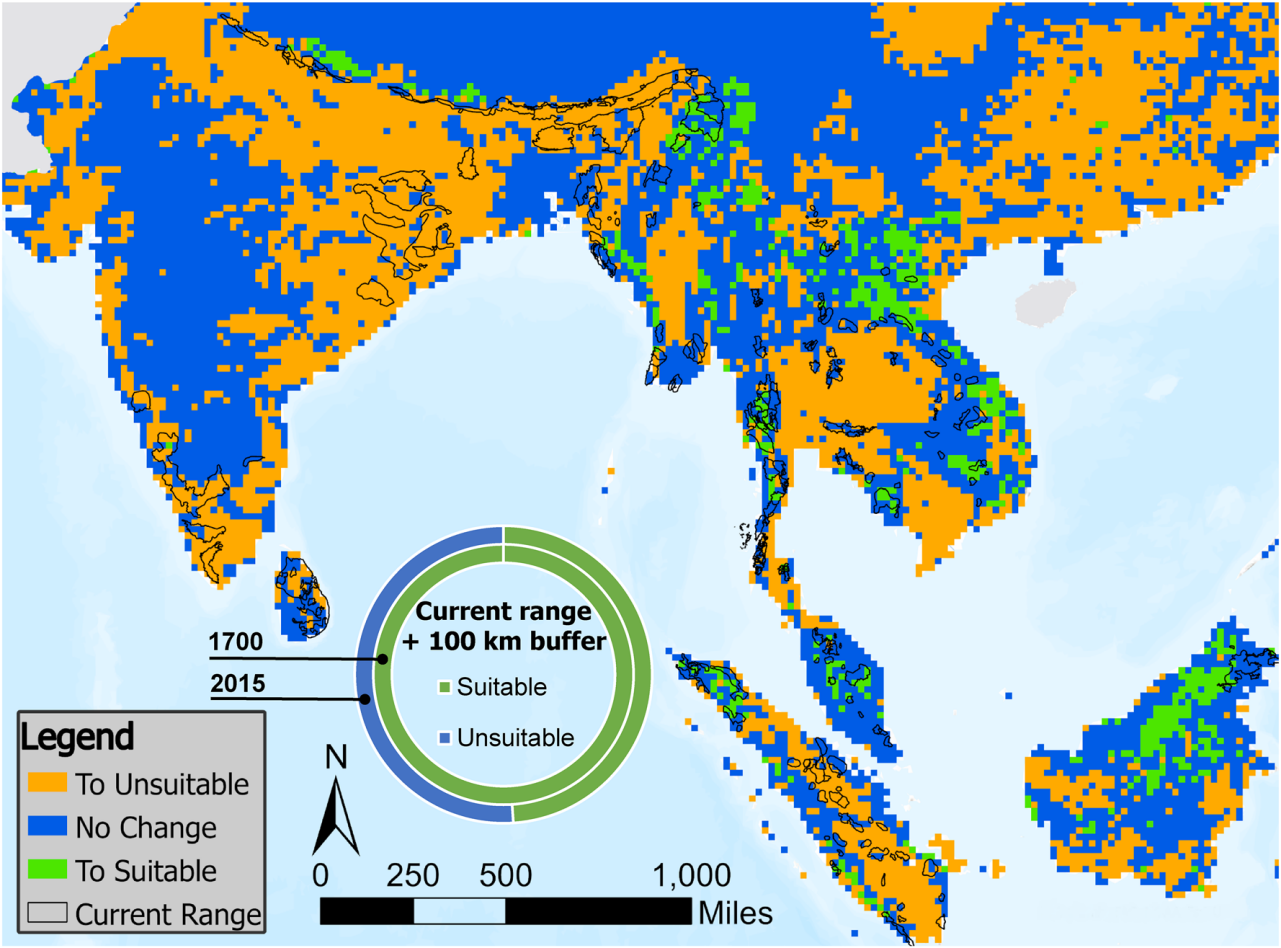
Loss and gain in suitable habitat across the range between 1700 and 2015. Masked areas (Pakistan, Hainan Island) have been excluded from analysis. Overall, 64.2% of the total area converted from suitable to unsuitable in this period, with 38.6% occurring within the current range (Table 3). Habitat gains largely occurred outside the current range. Donut chart shows that 100% of area within 100 km of the extant range was classified as suitable in the year 1700, but dropped to 48.94% by 2015 (see Figure S3 for timecourse).

Percentage of primary forest cover was less important for the LUH2 model than elevation, forested and non-forested primary and secondary lands, croplands, pastures and wood harvest activities (Table 1). The extent of ‘suitable’ habitat shows a significant decline (*t* test, *p* < 0.01 one-tailed). By the year 1700, 100% of the area within 100 km of the current range was still classified as suitable for elephants but as of 2015 the total extent of suitable area had decreased by 64% (− 3.36 million km^2^; Fig. 2). Over 38% of this loss occurred within the current range (Table 2). Average patch size fell by 83%, from 99,000 to 16,000 km^2^ and amount of area occupied by the largest patch (LPI) decreased 83% from 4 million km^2^ (~ 45% of total area) to just over 54,000 km^2^ (~ 7.5% of total area). The landscape contagion index nearly doubled (Fig. 2). Mainland China, India, Bangladesh, Thailand, Vietnam and Sumatra each lost more than half their suitable elephant range, where the greatest occurred in China (− 94%) and India (− 86%, Table 2). Bhutan, Nepal and Sri Lanka lost more suitable habitat inside than outside current elephant range (Table 2). Trends in Lao PDR and Malaysia showed a net gain, but not necessarily in areas within the current range (Table 2 and Fig. 3). Borneo appears to have experienced habitat restructuring rather than decline (Figs. 2, 3). Animations of the changes between 1700 and 2015 are provided in supplementary videos SV1 and SV2.

**Table 1:** LUH2 predictors with relative contributions > 1%.

Variable	Variable contribution (% change in AUC)	Permutation importance
C3 nitrogen-fixing crops	29.8	5.6
SRTM digital elevation	17.3	12.8
Potentially non-forested secondary land	9.9	10.7
Non-forested primary land	8.2	9.8
C3 annual crops	8.1	16.3
C4 perennial crops	6.5	4.9
Managed pasture	5.1	8.5
C3 perennial crops	4.1	0.3
Wood harvest area from secondary mature forest	3.2	5.8
C4 annual crops	2.1	1.8
Primary forest	1.9	5.9

Variables are ordered from most to least influential, noting that their contribution can be driven by either positive or negative associations. See Table 4 in methods for complete list of variables.

**Table 2:** Change in suitable habitat area by region from years 1700–2015.

	Suitable current area (km^2^)			Suitable potential area (km^2^)			Total suitable area (km^2^)		
	1700	2015	% change	1700	2015	% change	1700	2015	% change
Mainland China	1986	135	− 93.2	1,119,258	65,054	− 94.2	1,087,183	65,189	− 94.2
India	216,207	82,793	− 61.7	1,439,320	145,561	− 89.9	1,655,527	228,354	− 86.2
Bangladesh	6322	1770	− 72	37,725	10,634	− 71.9	44,046	12,405	− 71.8
Thailand	40,449	31,303	− 22.6	439,964	127,028	− 71.2	480,413	158,331	− 67.0
Vietnam	523	515	− 1.4	196,259	80,923	− 58.8	196,781	81,439	− 58.6
Indonesia (Sumatra)	45,252	27,507	− 39.2	317,636	123,278	− 61.2	362,888	150,785	− 58.5
Indonesia (Borneo)	826	928	12.3	428,709	282,061	− 34.2	429,535	282,989	− 34.1
Myanmar	32,026	36,591	14.3	289,533	181,179	− 37.4	321,559	217,770	− 32.3
Cambodia	7904	12,508	58.3	147,795	102,867	− 30.4	155,699	115,374	− 25.9
Nepal	12,086	4750	− 60.7	44,333	37,905	− 14.5	56,419	42,655	− 24.4
Sri Lanka	31,654	22,603	− 28.6	24,097	19,622	− 18.6	55,750	42,225	− 24.3
Bhutan	2033	1148	− 43.6	5,560	5,126	− 7.8	7593	6273	− 17.4
Lao PDR	16,507	17,716	7.3	148,922	159,843	7.33	165,429	177,558	7.3
Malaysia (Peninsular)	7796	10,682	37.0	95,029	105,418	10.9	102,825	116,100	12.9
Malaysia (Borneo)	7216	12,007	66.4	100,023	161,316	61.3	107,239	173,323	61.6
Totals	428,787	262,956	− 38.7	4,834,163	1,607,815	− 66.8	5,228,886	1,870,770	− 64.2

Areas are in km^2^, ordered from ranges that experienced the greatest loss to those with the greatest gain in total suitable area. “Suitable current area” refers to the current range, i.e. areas known to still contain elephants by the year 2000, whereas “Suitable potential area” refers to area that is outside the current range, where it is unknown whether elephants were ever present. The amount of suitable area within current range is 14% of the total suitable area.

### Present-day suitability versus distribution

Only 48.6% of the current range was found to be suitable by 2015 (Figs. 2, 3, S3 and Table 3). Of all the areas classified as suitable, just 14% actually occurs within the current range (Table 3). India has the largest proportion of the current range, but only about a third of it was classified as suitable by the year 2015. Sri Lanka and Malaysian Borneo appear to have estimated population sizes that are more than twice what would be expected relative to their share of the current range, with around 63% of the range in Sri Lanka and 95% of that in Borneo qualifying as suitable (Table 3). On the other hand, Lao PDR, Thailand and Myanmar have much lower estimated populations sizes than expected based on their share of the current range, despite approximately 79%, 60% and 51% of these ranges respectively being suitable. Most remaining range in Vietnam (98%) and Indonesian Borneo (100%) is suitable but extremely small, together accounting for just 0.27% of the total.

**Table 3:** Elephant population sizes relative to available range.

Range	Wild elephant population^a^	% of total population	Total current range (km^2^)^b^	% of total current range	Suitable habitat in 2015 (km^2^)	% of current range suitable in 2015	Range rank, population	Range rank, area	Rank ratio
Sri Lanka	5879	13.4	36,196	6.7	22,603	62.5	2	5	2.50
Malaysia (Borneo)	2268	5.1	12,589	2.3	12,007	95.4	4	9	2.25
China (Yunnan)	186	0.4	2362	0.4	135	5.7	11	13	1.18
Malaysia (Peninsular)	1450	3.3	13,413	2.5	10,682	79.6	6	7	1.17
Indonesia (Borneo)	167	0.4	928	0.2	928	100.0	12	14	1.17
Bangladesh	325	0.7	6770	1.3	1770	26.1	10	11	1.10
India	27,000	61.4	239,056	44.1	82,793	34.6	1	1	1.00
Indonesia (Sumatra)	2600	5.9	56,033	10.4	27,507	49.1	3	3	1.00
Vietnam	97	0.2	527	0.1	515	97.7	15	15	1.00
Cambodia	425	1.0	12,975	2.4	12,508	96.4	9	8	0.89
Bhutan	105	0.2	2424	0.5	1148	47.4	14	12	0.86
Nepal	126	0.3	12,178	2.3	4750	39.0	13	10	0.77
Lao PDR	700	1.6	22,494	4.2	17,716	78.8	8	6	0.75
Thailand	1000	2.3	52,415	9.7	31,303	59.7	7	4	0.57
Myanmar	1619	3.7	71,281	13.1	36,591	51.3	5	2	0.40
Totals	43,947	100	541,640	100	262,956	48.6	–	–	–

“Total current range” refers to the extent of range in which elephants were thought to be present in the 2000s. “Current Range Suitable in 2015” refers to the amount of this current range still classified as suitable habitat by the year 2015 under the LUH2 model. Ranges are ranked by the percentage of the global elephant population found within them as well as the percentage of global range they encompass (for China, this is limited to Yunnan province only). They are ordered by rank ratio, which is the area rank (based on % of total current range) divided by the population rank (based on % of total population). A ratio close to 1 indicates that the population size is proportional to the share of the current range within that region, higher ratios indicate populations are larger than expected on the basis of available range, and lower ratios indicate the opposite.

^a^From Fernando and Pastorini, 2011. Note that these population estimates reflect the time frame relevant to the datasets used in analyses rather than the most current estimates, which may have changed.

^b^Calculated from Hedges et al.^85^ (Fig. 1).

## Discussion

We find that after several centuries of relative stability, nearly two-thirds of habitat suitable for elephants within the 13 elephant range countries declined within the past 300–500 years. A gradual negative trend in the extent of suitable habitat commences as early as the 1500 s, but shows marked acceleration during the 1700s. Whereas all of the area (100%) within 100 km of the current range was classified as suitable in 1700, less than half (48.6%) of it was classified as suitable by the year 2015. Change in the Largest Patch Index (LPI) signifies that in the year 1700 an elephant might hypothetically have been able to traverse as much as 45% of the “suitable” area without interruption, but by 2015 this was down to just 7.5%. These include two of the top three most threatened ecoregions in the world, the tropical dry forest and tropical/subtropical grasslands and savannahs^16^. Our results corroborate genetic studies suggesting that although elephants are capable of dispersal over long distances, their gene flow may now be limited among populations that were connected until relatively recently^26,27^.

It has been shown that up to 90% of tropical woodlands were inhabited and shaped by human societies over the past 12,000 years, opposing the view that “human transformation of terrestrial nature is mostly recent and inherently destructive.”^28^ Given the ecology of elephants and long history of anthropogenic activity across these landscapes, the losses we report are unlikely to represent areas of pure “wilderness” or primary forest, but rather a mixture of habitat types, including those with some degree of human management^28,29^. Notably, both the extent of habitat loss and increases in habitat fragmentation, including of biomes such as forests^6,7^, are far greater than would be evident from analysis of the past century alone (Fig. 2), and may have commenced as early as the fifteenth century in some parts of the range^30^. Our results underscore that current trends should be seen as an extension of those that began during the colonial era, which was accompanied by the introduction of new value systems, market forces, and governance policies into continents beyond Europe^30,31^—including, but not limited to, forest biomes.

The presence of elephants in suboptimal habitat today likely reflects a lag between land-use changes and elephant population responses^32,33^. Elephants may no longer be able to disperse into some areas, nor persist where there has been heavy offtake in the past^34,35^. Conversely, in the absence of overharvest, populations may persist for centuries despite gradual demographic collapse^36^. The lack of adequate habitat in over half the current elephant range suggests a high potential for negative interactions with people in these areas, which deserves closer examination.

### Regional trends

Trends in South Asia are largely driven by India and Sri Lanka, which contain the largest remaining wild populations (Table 1). Strikingly, although our training data did not include locations from elephant range near the Eastern Ghats in central India (we could not verify their occurrence in habitat patches of appropriate scale for the LUH2 predictor variables), the model identified these areas as containing suitable habitat in the past but not the present (Figs. 2, 3). Both countries were transformed by colonial road-building and logging, during which elephants and other wildlife were eradicated from higher elevations and lowland rainforests which were converted to plantations and settlements^37^, so much so that current elephant distributions in Sri Lanka more closely match areas classified as suitable habitat in the year 1700 than 2015 (^38^ and this paper, Fig. 2). A substantial portion of present-day elephant range includes mosaics with substantial human activity^38,39^.

The relationship between people and nature today is complicated by the ongoing expansion of population centers, agriculture sectors, and extractive economic activity that places additional pressure on forest resources^40^. Forest/agriculture boundaries also encourage conflict with wildlife, especially in the wake of fresh deforestation^41–43^. A study in central Assam found that conflict with elephants dramatically increased in the 1980s, corresponding to a drop in forest cover below 30–40%^44^. Likewise, another study around the Nilgiri Biosphere Reserve found that deforestation was associated with increases in negative incidents with elephants^45^. In Sri Lanka, studies relating to LULC changes to official records of incidents with elephants over more than a decade found a strong link between LULC trends and increased conflicts^46,47^, with up to 98% occurring within 1 km of a recent land-use conversion^46^. Hotspots of conflict developed especially following the end of the civil war in 2009, which was accompanied by increased infrastructure projects and other development initiatives^47^. From the perspective of our study, conflicts between people and elephants may be viewed as symptomatic not only of recent, proximate land-use modifications, but also the long-term legacies of land management paradigms put in place over the past few centuries.

This longer perspective also contextualizes another recent study in Nepal that documented changes in forest cover between 1930 and 2020 using topographic maps and satellite imagery^48^. It was found that core elephant ranges consisting of large forests decreased by 43.08%, while the number of smaller patches increased. The study reports that 21.5% of elephant habitat was lost overall during this period. The apparent increase in fragmentation is consistent with our observations, but we found there to be a 60.7% decrease in suitable habitat within the known elephant range in Nepal since the 1700s and an overall decrease in suitable habitat by 24.4% throughout the country (Table 3). Ram et al.^48^ also point out the association between areas of habitat fragmentation and negative encounters, including human fatalities, as elephants move between patches through areas of human activity. The extent of habitat loss, which includes non-forested environments, is therefore likely to be greater than can be quantified directly using data on forest cover change over the past century but negative incidents may be most prevalent in areas of relatively recent deforestation as remnant elephant populations continue to be displaced.

In Southeast Asia, the disappearance of highly suitable habitat in what is now central Thailand is particularly striking (Figs. 2, 3), much of it occurring between 1950 and 1990 (supplementary videos SV1 and SV2). These areas now contain mostly cropland. This therefore reflects not only historic timber extraction and associated land-use conversions (Thailand was not itself colonized), but also the more recent “Green Revolution” expansion of industrial agriculture. Although expanses of forest remain in Thailand and Myanmar, both have lower estimated elephant populations than expected based on their share of the current range (Table 1). This might be driven by habitat quality^35^ or high rates of historic offtake for both the timber and tourism industries^49–51^. As a result, captive elephants now likely outnumber wild elephants in both countries^35,49^. Myanmar had also been experiencing high rates of poaching for skins^52^. Yet both countries have interest in re-establishing wild elephant populations into areas of available habitat. The proximate and long-term drivers of elephant decline due to habitat quality, hunting, and conflict with people must be taken into consideration in making such plans^53^.

Two of the most critically endangered elephant populations are found in Sumatra and Vietnam. Although nearly 98% of the current range in Vietnam is classified as suitable (Table 1), the extent of this range is extremely small (527 km^2^). Both cases require concerted efforts to recover habitat and re-connect isolated wildlife populations through ecosystem-level management. Our results align with other studies^32,54^ showing that primary forest is less important for elephants than other ecosystem types (Table 2). One feature of elephant habitat may be seasonally rotating swidden (shifting) cultivation^55^, which was traditionally practiced widely in these regions, but is now in decline across many parts of Asia^55–59^. Shifting cultivation is predicted to entirely disappear from Asia within the coming century^60^. This is a cause for concern not only from the standpoint of potential loss of traditional ecological knowledge, but also in terms of the ecological regimes such practices represent, which may benefit certain wildlife and encourage more biodiversity at larger spatial scales than the permanent forms of agriculture and plantations which replace them^55,58^. The impact of shifting cultivation on deforestation itself is a topic of much investigation, and it is increasingly clear that the relationship is complex, being scale- and density-dependent^56,58^. Nevertheless, maintenance and restoration of such regimes to some degree may not only facilitate habitat recovery and connectivity, but also be beneficial for agrarian communities.

LULC change is both a cause and a consequence of human and wildlife displacements. Our results indicate that areas of suitable habitat for elephants have not merely decreased, they have also redistributed (Fig. 3). The movements of people as much as elephants merge ecological events with social, economic and political issues. In 2018 there was rapid, large-scale disruption of a trans-boundary elephant corridor at Cox Bazar between Bangladesh and Myanmar with the settlement of Rohingya refugees^61^. Conversely, range shifts by elephants can introduce challenges for human communities that have little experience with elephants. In 2020, the long-range movement of a small herd of elephants out of a protected area in Yunnan province, China, generated global headlines^62^. Their foray lasted for more than a year and covered more than 500 km, with over 150,000 people being evacuated from their path in 2021^63^. Such attempted dispersal events out of protected areas suggest habitat pressure for remnant elephant populations and highlight the challenges of moving to suitable habitat outside extant ranges. Interestingly, our results suggest that suitable habitat for elephants persisted in China long after local populations went extinct.

Given the depletion and fragmentation of suitable habitat, as well as elephants’ preference for secondary and regenerating habitat, attempts at dispersal outside the current range might be expected^23^. However, these new agricultural landscapes, unlike agroecological systems of the past, are characterized by a greater degree of human antagonism towards wildlife which must be accounted for both in managing wildlife and land-uses. But protected areas in Asia tend to be small^64^ and biased toward rugged terrain as well as higher elevations^65^, thus they cannot fully accommodate elephant populations. If remnant populations are to survive, the practice of driving them into ever-shrinking and marginal habitat must be replaced with attempts to adequately identify and connect areas of suitable habitat. Our results identify such areas at coarse scale, but more refined characterizations based on both ecological and human considerations are needed (see study limitations, below).

### Study limitations and possible extensions

There are a number of reasons why the actual distribution of a species may not match its modelled niche, such as dispersal limitations or overharvest (i.e. hunting and capture, see also^66^). We have already discussed several of these issues with respect to elephants. Our intent was to have this species serve as surrogates for the ecosystem types they could potentially occupy and we believe these results are robust out of several considerations. First, our finding that all of the area within 100 km of the current range could be considered suitable habitat by the 1700s, including regions that were not originally sampled, gives us confidence that the sampling locations and the resultant model adequately capture essential requirements of the species. Second, our results closely match a review of 4018 terrestrial mammals showing that on average 48.6% of a species’ range could be classified as “suitable” on the basis of their preferred habitats^3^ and corroborates previous studies showing that habitat adequate for elephants is being lost even inside protected areas^48,67,68^. Finally, our results align with the finding that terrestrial biomes globally underwent a transition from being “mostly wild” to “mostly anthropogenic” in the period between 1700 and 2000^29^.

Other limitations concern the definition of what actually constitutes “suitable” habitat for the species. Ecological niche models typically rely on relating the species of interest (i.e. occurrence, behavior) to ecological covariates. They exclude at least two important considerations. First, habitat characteristics offer a limited view of which areas may support a particular species in the absence of demographic data. Animals may be attracted to locations that promote harm to them; these are known as ecological traps^69^. As an extreme example, some elephants may routinely use garbage dumps or tourist outposts where they are fed, but these do not constitute appropriate sampling locations. We were conservative in our sampling, excluding the possibility that elephants might potentially flourish in some (present-day) human-dominated landscapes. This decision is underpinned by the second set of considerations: human perceptions and behavior. A species generally cannot flourish on a landscape that is otherwise ideal if people actively exclude or suppress it. Indeed, present-day land-use and development policies largely ignore the potential for negative human-wildlife interactions. For instance, governmental subsidies for irrigation infrastructure and use-it-or-lose-it policies of land tenure promote cultivation of conflict-prone food crops such as rice, fruits and vegetables in and around wildlife habitat (SdS, personal observations). Such policies will likely exacerbate conflict and drive elephant population declines, unless national development agendas are re-aligned with countries’ stated commitments to biodiversity conservation. Given these realities, for the purposes of our study, we have chosen to expressly avoid characterization of intensively cultivated agricultural/plantation zones regardless of their potential for accommodating the species. Certain human-modified landscapes (“working landscapes”) could play a pivotal role for elephants as well as other wildlife in the future by partially compensating habitat loss and fragmentation^70^, *but only if* sustainable paradigms of coexistence are achieved^17,71^.

Conversely, we must also avoid mistaking the present management of a landscape with the practices that gave rise to it. We reiterate that although human activities are now limited in the locations we sampled, these areas were very likely also shaped by people preceding and during the time scales being considered here^28^. It is important to acknowledge that pre-colonial societies with legacies of sustainable resource management have been and continue to be displaced, including through the creation of protected areas, owing to a land-use paradigm of separating human/nonhuman spaces^72–74^. Attempts at habitat “restoration” or reconciliation of human land-uses with elephants and other wildlife requires an honest reckoning with issues of social and environmental justice with respect to the rights of historically marginalized communities in modern economies and governance structures^75^. Exploring the relationship between past land management practices and the distributions of elephant ecosystems would be a useful direction for future studies from the perspectives of both ecological and social policy.

Finally, the datasets underlying our results (the LUH2 variables) use relatively coarse resolution and are themselves models based on assumptions concerning land-use transitions rather than direct measurements and observations. In fact, results based on the LUH2 reconstructions may be more conservative than models using finer resolution data that make fewer assumptions. Winkler et al.^8^, for instance, present global land-use change models for the period between 1960 and 2019 which combine various high-resolution remote sensing and statistical datasets at a spatial resolution of 1 km and find estimates of change to be as much as four times higher than those using other data products^8^. Specifically, in comparison to the LUH2, the mean annual change was just over twice as high. If a similar extrapolation can be made retrospectively, it suggests that rates of loss may be *even greater* than our results show. Closer examination of fine-scale mechanisms behind trends in particular land-use types obtained by merging spatially-explicit anthropological and archeological data may offer additional insights into the past.

Likewise, more work is needed to understand possible changes in suitable habitat under future scenarios of land-use. One study using fine-resolution (1 km^2^) LULC datasets for India and Nepal predicted a loss of 41.8% of the available habitat over this century^76^. The LUH2 datasets are also available for models of future scenarios of climatic and socioeconomic change, which offer a means to predict how suitable habitats may continue to shift at broader spatial scale. Such assessments, in conjunction with an understanding of site-specific histories would offer much-needed guidance for the management of elephants and our shared ecosystems.

## Methods

### Elephant occurrence

**Figure 4:**
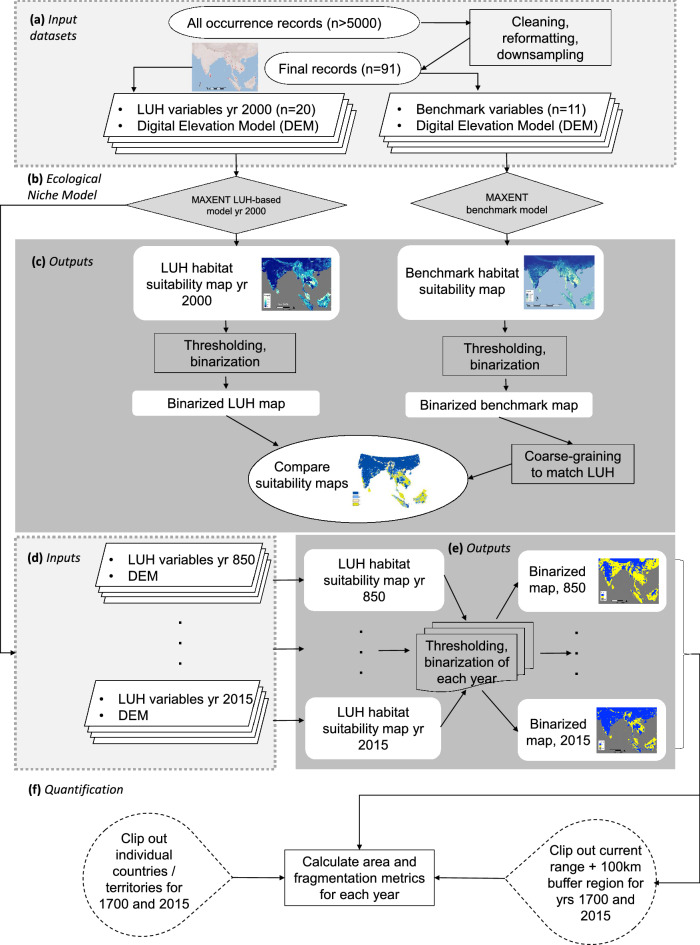
Flow chart of study. (**a**) Input variables used in training and initial evaluation include the SRTM digital elevation model (DEM) and 20 LUH2 variables for a total of 21 variables; input variables for benchmark variables include DEM and 11 other datasets (see Table S1). (**b**) Ecological niche model constructed. (**c**) Comparison of model results for the whole range and by country/territory to evaluate degree of agreement (see supplementary text for results). (**d**) Model runs for each selected year. (**e**) Binary maps created for each year. (**f**) Quantification of extent and fragmentation of habitat for the entire region across each time point, by country/territory for the years 1700 and 2015, and for areas within 100 km of the extant range for 1700 and 2015.

A schematic of the work process is given in Fig. 4. Elephant occurrence locations were initially compiled from the Global Biodiversity Information Facility (https://www.gbif.org/), Movebank (https://www.movebank.org/) and published literature^77–79^ as well as data contributed by the authors based on direct sightings, data logged via tracking devices, and camera traps (n > 5000 locations). Records were first checked visually for irrelevant points (e.g., occurrences outside natural continental range, from GBIF) then refined to include locations representing ecosystems where the species could conceivably flourish, including but not exclusively limited to protected areas. We resist labelling these landscapes as “natural” or “wilderness” areas in recognition that most, if not all, landscapes are likely to have had some degree of human influence in the near or distant past^28^. For instance, we included selectively logged forest because secondary or regenerating forest can support elephants with potentially little conflict with humans and many forests have some history of management. We also included sanctuaries containing reservoir systems of both ancient and recent (twentieth century) creation, where large elephant populations now exist (Fig. 1). We excluded intensively managed croplands and plantations given their high potential for negative interactions with people^17,36^.

To minimize sampling bias that could result in model overfitting, we further subsampled data to cover the full distribution as widely as possible while eliminating redundant points located within any particular landscape. For instance, thousands of potential redundancies from collar-based tracking datasets were removed by using only one randomly selected data point per individual, per population or landscape. Outliers from the remaining points were removed using Cooks’ distance^80^ to eliminate locations that could represent potential errors. The final dataset consisted of 91 occurrence points spanning the years 1996–2015 which served as training data (Fig. 1), where all data other than from GBIF and cited literature were contributed by the authors or individuals listed in acknowledgments. QGIS and Google Earth Pro were used to initially visualize and process the data.

### Predictor variables

We used the Land-Use Harmonization 2 (LUH2) data products^25^ as our environmental variables. The LUH2 datasets provide historical reconstructions of land use and land management from 850 to 2015 CE, at annual increments. The LUH2 data products were downloaded from the University of Maryland at http://luh.umd.edu/data.shtml (LUHv2h “baseline” scenario released October 14th 2016). They contain three types of variables gridded at 0.25° × 0.25° (approximately 30 km^2^ at the equator): state variables describing the land-use of a grid cell for a given year, transition variables describing the changes in a grid cell from one year to the next, and management variables that describe agricultural applications such as irrigation and fertilizer use, totaling 46 variables. Of these, we selected 20 variables corresponding to all 3 types (Table 4), which were expected to be relevant to elephant habitat use based on knowledge of the species’ ecology^21,22,32,81^. Using ArcGIS 10 (ESRI 2017) we extracted each variable between 850–1700 CE at 25-year increments, and annually between 1700 and 2015. We separately obtained elevation from the SRTM Digital Elevation Model (Table S1).

**Table 4:** Included land-use harmonization (LUH) variables.

States (units: fraction of grid cell)	Transitions between land use states (units: fraction of grid cell per year)	Management—irrigation (units: fraction of crop area)
primf: forested primary land	primf_harv: wood harvest area from primary forest	irrig_c3ann: irrigated fraction of C3 annual area
primn: non-forested primary land	primn_harv: wood harvest area from primary non-forest	irrig_c3per: irrigated fraction of C3 perennial area
secdf: potentially forested secondary land	secmf_harv: wood harvest area from secondary mature forest	irrig_c4ann: irrigated fraction of C4 annual area
secdn: potentially non-forested secondary land	secyf_harv: wood harvest area from secondary young forest	irrig_c4per: irrigated fraction of C4 perennial area
pastr: managed pasture		irrig_c3nfx: irrigated fraction of C3 nitrogen-fixing area
c3ann: C3 annual crops		flood: flooded fraction of C3 annual crop area
c3per: C3 perennial crops		
c4ann: C4 annual crops		
c4per: C4 perennial crops		
c3nfx: C3 nitrogen-fixing crops		

Note that although wood harvest rates are labelled as “transition” variables representing land-use conversions between years, they quantify the percentage of a pixel harvested within a given year and so can be used alongside the other two types of annual layers.

### Data analysis and benchmarking

We limited the geographic extent of all analyses to the 13 range countries in which elephants are currently found. We used MAXENT, a maximum entropy algorithm^82^, to model habitat suitability using the ‘dismo’ package in R (R Core Team^83^). Resulting raster files were binarized in ArcGIS into suitable and unsuitable habitat with a pixel size of approximately 20 km^2^ as a cutoff threshold. As there is no commonly accepted threshold type^84^, to ensure that the specific choice of threshold did not affect the observed trends, we initially used three possible thresholds: 0.237, representing ‘maximum test sensitivity plus specificity,’ 0.284 corresponding to ‘maximum training sensitivity plus specificity,’ and 0.331 representing ‘10th percentile training presence’. Unless otherwise stated, for subsequent analyses we show only results using the threshold of 0.284, where everything below this threshold was classified as ‘unsuitable’ and everything above it was classified as ‘suitable’. The resulting binary maps were re-projected using the WGS84 datum and an Albers Equal Area Conic projection.

To establish whether a model using the LUH2 variables yields reasonable predictions of habitat suitability for elephants, we first compared the result for the year 2000 to a prediction based on other, higher resolution benchmark variables, including climate, terrain, land-cover, and human and livestock densities (n = 12 variables, Table S1). The details of this comparison and results are provided in the Supplementary Information ("Introduction" section, Figures S1 and S2), which showed the two sets of predictions to be in agreement for over 80% of pixels (including 89% of pixels within the current elephant range). The LUH2 prediction was slightly more conservative (i.e. classifying fewer pixels as suitable) than the benchmark prediction. The LUH2 model was then applied to all focal years between 850 and 2015.

Polygons representing the known elephant range were digitized from Hedges et al.^85^ from the category labelled as “active confirmed”. We refer to the areas within these polygons as “current range,” and refer to areas outside them as “potential range”. We compared the total extent of suitable habitat within and outside the current elephant range, quantifying changes over time. Country-level analyses were conducted for all countries except Indonesia and Malaysia where the Bornean and Sumatran ranges were treated separately in recognition of the distinct subspecies in these two regions. We included the entirety of Borneo because both genetic studies and geological history allow for the possibility that elephants could have been natively distributed throughout the island^86,87^, and there are no present-day physical barriers to dispersal on the island. However, in visualizing results we distinguish the currently accepted range. We ranked each region based on the percentage of the current range within that region as well as the proportion of the estimated elephant population found within it, and calculated the ratio of these ranks (Table 3).

We calculated the total change in extent of suitable habitat by subtracting the area of suitable habitat available in 2015 from the area available in 1700, as major changes were observed within this period. We also specifically quantified the percentage of suitable habitat found within a 100 km buffer of the current range polygons in both years. We then calculated fragmentation statistics (Table S2) using the program FRAGSTATS v.4.2^88^. These metrics characterize changes to the spatial configuration of habitat in addition to their absolute extent. We used a ‘no sampling’ strategy with the search radius and threshold distance set to 61 km (approximately three pixel lengths) based on the movement and dispersal capacity of elephants^89,90^. We tested for the significance of change in the extent of suitable habitat before and after a major inflection point in the curve (see results) by calculating the change over 100-year increments and comparing average slope using a two-sample *t* test with unequal variances.

## Supplementary Information

Supplementary Information 1.

Supplementary Video 1.

Supplementary Video 2.

## Data Availability

Data supporting the results is available on the DRYAD data repository at 10.6076/D1P305.
