# Supplementary material for: Land-use change is associated with multi-century loss of elephant ecosystems in Asia
Source: Sci Rep. 2023 Apr 27;13:5996. doi: 10.1038/s41598-023-30650-8 (PMC10140153; doi:10.1038/s41598-023-30650-8)

<u>Land-use Change Is Associated With Multi-Century Loss Of Elephant Ecosystems in Asia</u>

Supplementary Material

1. Comparison of habitat suitability predictions using Land-Use Harmonization (LUH) variables vs. other contemporary benchmark variables.

1.1 Data analyses

To evaluate whether a model derived from LUH2 variables (Table 4, main text) produce acceptable habitat suitability predictions, we compared it with a model based on *benchmark variables* (Figure 4, main text). For the LUH2 prediction, we used the year 2000 as our base model and comparison year because it is near the midpoint of the timeframe over which elephant occurrence data were available (Figure 1, main text). The benchmark variables included widely-used, higher resolution geospatial data spanning the years 2000-2005, thus also overlapping with our occurrence data. These variables were resampled to match the variable with the coarsest resolution (0.5°) before constructing the MAXENT model. Both the LUH model and benchmark model additionally included the SRTM digital elevation model as an input feature. Raster files were then binarized in ArcMap into suitable and unsuitable habitat for both sets of variables, with a cutoff threshold corresponding to 'maximum training sensitivity plus specificity' (see Methods). For the benchmark model this value was 0.350, for the LUH model it was 0.284; everything below the threshold was thus classified as 'unsuitable' while everything above was classified as 'suitable' for subsequent analyses. To compare the results derived from both sets of predictors, the results of the benchmark variables were again resampled to match the coarser resolution of the LUH dataset.

Table S1. Benchmark environmental predictor variables for comparison model. Contribution and permutation importance for the MAXENT model are listed for those variables with relative contributions >1% change in AUC, with variables ordered from most to least influential.

Variable	Variable contribution (% change in AUC)	Permutation importance	Time period used	Spatial resolution	Source
Bioclim seasonality	46.9	41.4	2000	~1 km	http://www.worldclim.org/current (Hijmans, 2005)
FAO sheep and goat density	16	15.4	2005	0.05 Decimal Degree, ~5 km	http://www.fao.org/geonetwork/srv/en/main.search?extended=off&remote=off&any=glw+12717&themekey=&to=&from=&siteId=&hitsPerPage=10 (Robinson, 2014)
Percent tree cover	15	0.2	2000	250 m	Matt Hansen, University of Maryland https://earthengine.google.org/#detail/UMD%2Fhansen%2Fglobal_forest_change_2013
EarthStat cropland	5.4	7.1	2000	5 min (~10 km)	http://www.earthstat.org/data-download/ (Ramankutty, 2008)
SRTM digital elevation	4.6	7.1	2003	1 km	http://www.cgiar-csi.org/data/srtm-90m-digital-elevation-database-v4-1#citation (Jarvis, 2008)
Landscan human population	4.2	2.8	2009	1 km	http://web.ornl.gov/sci/landscan/landscan_documentation.shtml (Vijayaraj, 2008)
EarthStat pasture	3.5	0.9	2000	5 min (~10km)	http://www.earthstat.org/data-download/ (Ramankutty, 2008)
Annual mean temperature	2	2.3	2000	~1 km	http://www.worldclim.org/current (Hijmans, 2005)
Percent non-vegetated cover	1.9	21.8	2001	250 m	https://lpdaac.usgs.gov/dataset_discovery/modis/modis_products_table/mod44b (DiMiceli, 2011)
Slope			2003	1 km	Derived from SRTM DEM

Percent non-tree vegetation			2001	250 m	https://lpdaac.usgs.gov/dataset_discovery/modis/modis_products_table/mod44b (DiMiceli, 2011)
FAO cattle density			2005	0.05 Decimal Degree, ~5 km	http://www.fao.org/geonetwork/srv/en/main.search?extended=off&remote=off&any=glw+12713&themekey=&to=&from=&siteId=&hitsPerPage=10 (Robinson, 2014)

1.2 Results of comparison

We first compared predictions of habitat suitability under the benchmark model to those under the LUH model (Figure S1). After binarization, datasets were in agreement for 80% of pixels overall (>7 x 10^6 km^2) and 89% of pixels within the current elephant range. They were in agreement on over 80% of the area for 6 out of 13 countries (China, Bangladesh, India, Sri Lanka, Bhutan and Cambodia; Figure S2). Indonesia, Myanmar, Thailand and Vietnam had lower levels of agreement. The lowest agreement occurred for Indonesia (58% for the Sumatran area, 56% for the Bornean area) and Vietnam (53% of area). Areas of disagreement tended to occur at intermediate values, i.e., at the transition zones from suitable to unsuitable, reflecting loss of information in converting from graded to binary outputs. Slightly more pixels were classified as "suitable" under the benchmark model relative to the LUH model (Figure S1D), thus the latter was more conservative. Nevertheless, given the high levels of agreement overall, we considered the LUH model adequate for the purposes of this study.

Figure S1. Comparison of present-day habitat suitability modelled with the benchmark vs. LUH variables. In (A) and (B) blue represents less suitable areas whereas yellow represents more suitable areas. The masked areas (Hainan Island and part of Pakistan) were not included in analyses. C) 1=Pixel classified as unsuitable under both models; 2=Pixel classified as suitable under benchmark model and unsuitable under LUH model; 3=Pixel classified as unsuitable under benchmark model and suitable under LUH; 4=Pixel classified as suitable under both models. D) Histogram of pixel classifications in (C). Figures were generated in by S. de Silva & T. Wu in R, v. 3.5.0 (https://www.r-project.org/).

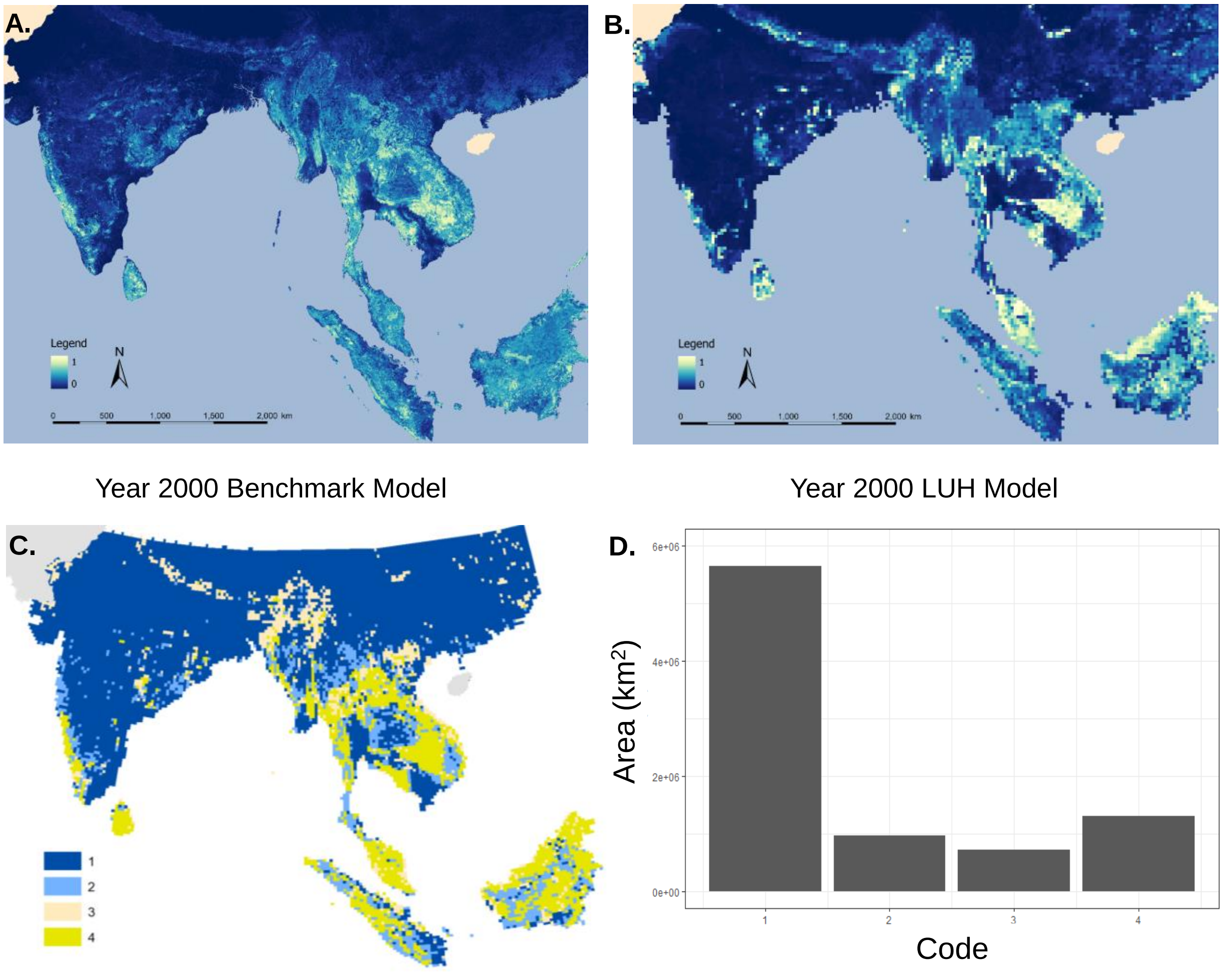

Figure S2. Degree of agreement between LUH and benchmark models upon binarization. 1=Pixel classified as unsuitable under both models; 2=Pixel classified as suitable under benchmark model and unsuitable under LUH model; 3=Pixel classified as unsuitable under benchmark model and suitable under LUH; 4=Pixel classified as suitable under both models.

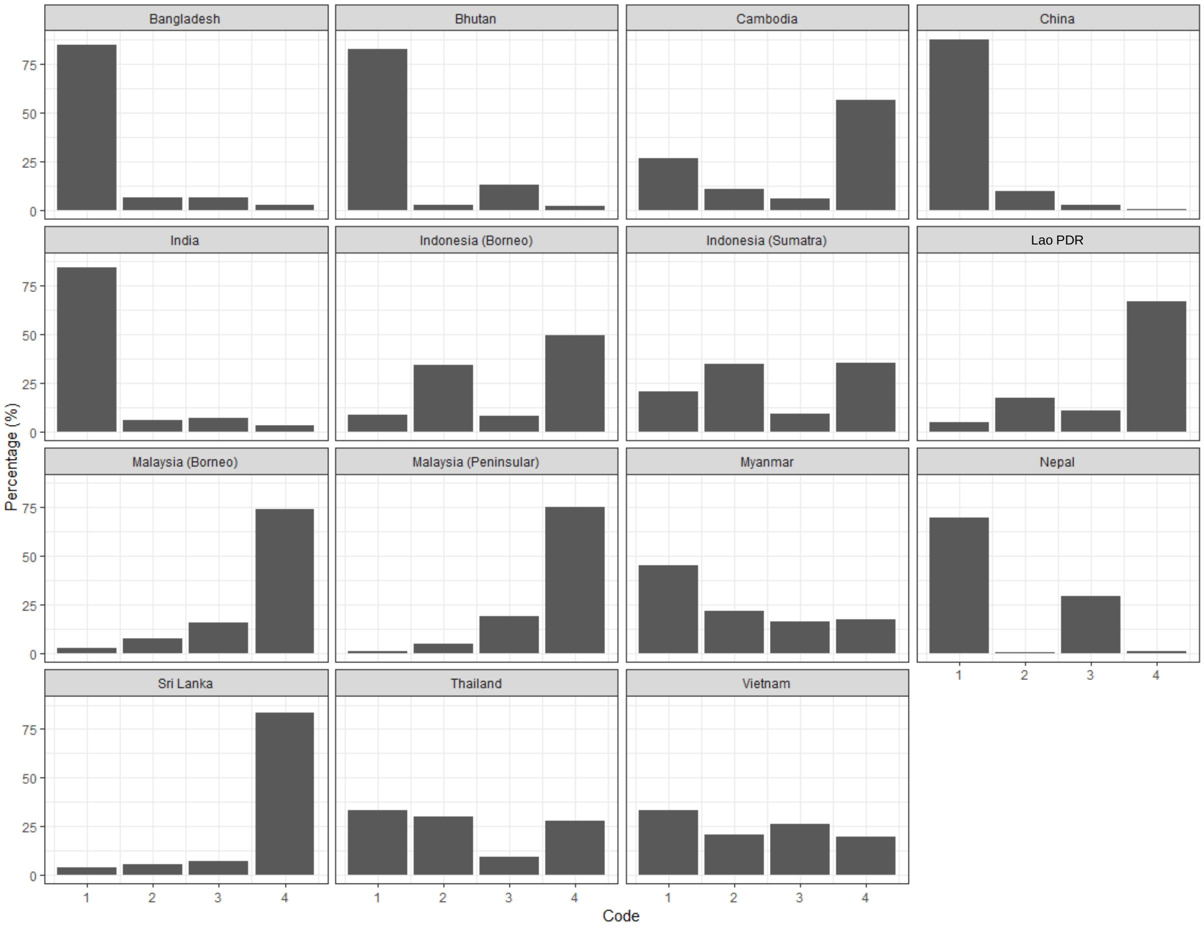

2. Additional Tables and Figures

Table S2. Summary of variables analyzed in FRAGSTATS. As indicated in bold, only results for total area, area-weighted mean patch size, largest patch index (class metrics), and contagion (landscape metrics) are shown as these most clearly and intuitively illustrate trends in habitat suitability and structure.

<table>
<tr><th>Patch metrics</th><th colspan="2">Class metrics</th><th colspan="2">Landscape metrics</th></tr>
<tr><td>Patch area (AREA)</td><td colspan="2">Total Area (CA/TA)</td><td colspan="2">Contagion (CONTAG)</td></tr>
<tr><td>Euclidean Nearest-Neighbor Distance (ENN)</td><td colspan="2">Percentage of Landscape (PLAND)</td><td colspan="2">IJI</td></tr>
<tr><td rowspan="21"></td><td colspan="2" rowspan="3">Largest Patch Index (LPI)</td><td rowspan="3">Proximity Index (PROX_?)</td><td>MN</td></tr>
<tr><td>AM</td></tr>
<tr><td>CV</td></tr>
<tr><td rowspan="5">Patch Area (AREA_?)</td><td>Mean (MN)</td><td colspan="2" rowspan="5">CONNECT</td></tr>
<tr><td>Area-Weighted Mean (AM)</td></tr>
<tr><td>Median (MD)</td></tr>
<tr><td>Range (RA)</td></tr>
<tr><td>Coefficient of Variation (CV)</td></tr>
<tr><td colspan="2">Perimeter-Area Fractal Dimension (PARFRAC)</td><td colspan="2" rowspan="13"></td></tr>
<tr><td rowspan="4">Contiguity Index (CONTIG_?)</td><td>MN</td></tr>
<tr><td>AM</td></tr>
<tr><td>Standard Deviation (SD)</td></tr>
<tr><td>CV</td></tr>
<tr><td rowspan="3">Euclidean Nearest Neighbor Distance (ENN_?)</td><td>MN</td></tr>
<tr><td>AM</td></tr>
<tr><td>CV</td></tr>
<tr><td colspan="2">Number of Patches (NP)</td></tr>
<tr><td colspan="2">Interspersion Juxtaposition Index (IJI)</td></tr>
<tr><td rowspan="3">Proximity Index (PROX_?)</td><td>MN</td></tr>
<tr><td>AM</td></tr>
<tr><td>CV</td></tr>
<tr><td></td><td colspan="2">Connectance Index (CONNECT)</td><td colspan="2"></td></tr>
</table>

Figure S3. Timecourse of habitat loss in and around current elephant range. Values represent three different binarization thresholds (see methods).

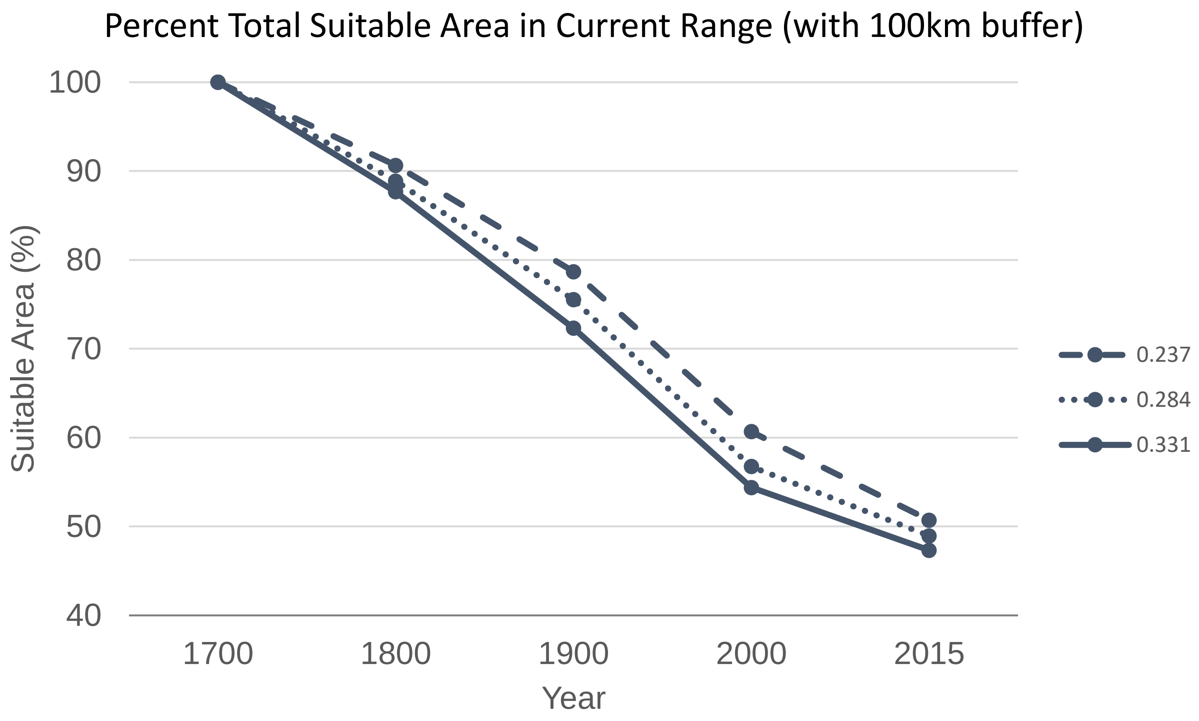

Supplement: Supplementary file 1 — Supplementary Information 1. [file 41598_2023_30650_MOESM1_ESM.pdf]
